# Weight reduction is not a major reason for improvement in rheumatoid arthritis from lacto-vegetarian, vegan or Mediterranean diets

**DOI:** 10.1186/1475-2891-4-15

**Published:** 2005-05-04

**Authors:** Lars Sköldstam, Lars Brudin, Linda Hagfors, Gunnar Johansson

**Affiliations:** 1 Department of Medicine, County Hospital, Visby, Sweden; 2 Department of Clinical Physiology, County Hospital, Kalmar, Sweden; 3 Department of Food and Nutrition, Umeå University, SE-901 87 Umeå, Sweden

**Keywords:** Rheumatoid Arthritis, therapy, diet intervention, lacto-vegetarian diet, vegan diet, Mediterranean diet, body weight

## Abstract

**Objectives:**

Several investigators have reported that clinical improvements of patients with rheumatoid arthritis (RA), from participating in therapeutic diet intervention studies, have been accompanied by loss of body weight. This has raised the question whether weight reduction per se can improve RA. In order to test this hypothesis, three previously conducted diet intervention studies, comprising 95 patients with RA, were pooled. Together with Age, Gender, and Disease Duration, change during the test period in body weight, characterised dichotomously as reduction or no reduction (dichoΔBody Weight), as well as Diet (dichotomously as ordinary diet or test diet), were the independent variables. Dependent variables were the difference (Δ) from baseline to conclusion of the study in five different disease outcome measures. ΔESR and ΔPain Score were both characterised numerically and dichotomously (improvement or no improvement). ΔAcute Phase Response, ΔPhysical Function, and ΔTender Joint Count were characterised dichotomously only. Multiple logistic regression was used to analyse associations between the independent and the disease outcome variables.

**Results:**

Statistically significant correlations were found between Diet and three disease outcome variables i.e. ΔAcute-Phase Response, ΔPain Score, and ΔPhysical Function. Δ Body Weight was univariately only correlated to ΔAcute-Phase Response but not significant when diet was taken into account.

**Conclusion:**

Body weight reduction did not significantly contribute to the improvement in rheumatoid arthritis when eating lacto-vegetarian, vegan or Mediterranean diets.

## Introduction

We have recently found that patients with rheumatoid arthritis (RA) improved significantly in disease activity from eating a modified Cretan Mediterranean diet (MD) [1]. Improvement was seen in 9 of 14 efficacy variables. The RA disease activity as measured by the "Disease Activity Score" DAS28 [2] was reduced by 13 %. In comparison the control group showed no change. The favourable outcome of the diet intervention group indicated that the MD had been suppressive to RA inflammation. We believe that the anti inflammatory effect of the MD was mediated by its different lipid profile [1] in conjunction with its high content of fresh fruits and green vegetables. However, at the end of the experiment the patients of the MD group, but not the control group, had lost 3 kg in weight (p < 0.001 between groups), although our ambition had been to prescribe a diet that was isocaloric compared to the patients' previous food intake. This unexpected weight loss raised the question whether a reduced energy intake, could have been yet another anti-inflammatory factor? That diet intervention studies may induce unexpected weight reduction to patients with RA is a finding shared by others [3-9]. Some researchers [7,8] have even reported a statistically significant correlation between change in body weight with change in arthritis measurements.

The aim of the present study was to investigate whether weight reduction could have anti inflammatory effect on RA. Data from three previously performed diet intervention studies were pooled for the analysis.

## Methods

### Design

Data from three different diet intervention studies, which we had previously conducted on patients with rheumatoid arthritis [1,3,4] were pooled and reanalysed.

Two studies (1^st ^and 3^rd^) had a prospective, randomised, and parallel design. The 2^nd ^was a prospective, cross over study. Nor the participating patients or the clinical outcome valuators were blinded with respect to the study designs.

### Patients

The pooled patient material comprises 95 Caucasians with RA according to the American College of Rheumatology (ACR) criteria from 1984. All but one had active disease. The three populations showed equal distributions with respect to sex, age, disease duration, functional capacity and stage of RA disease (Table 1).

**Table 1:** Baseline characteristics of patients who completed the trials.

	Diet group **(n = 60)**	Control group **(n = 42)**
Age (years)	54.5 (33–73)	57.0 (35–75)
Gender (M/F)*	11/49	7/35
Weight (kg)	72.1 (40–109)	69.4 (41–102)
Disease duration (years)	13.0 (0.5–59)	11.9 (2–35)

Footnotes to table:

Data are presented as mean (range) unless otherwise stated.

* Number of male and females, respectively

### The original studies

In the first study [3] 24 RA patients concluded the trial. 14 tested a lacto-vegetarian diet, and 10 were controls. Eight from the diet group reported less pain at the end of the experiment. As a group they lost 2.6 kg (p < 0.001) in body weight, but showed no statistically significant change in disease outcome measures. The control group stayed unchanged.

In the second study [4], which had a cross over design, 20 RA patients concluded the trial. All were initially followed for a control period of two or five months, 13 and 7 patients, respectively. Thereafter, all patients adopted a vegan diet for 4 months. During the vegan diet period the group of 20 patients obtained statistically significant reduction in pain score, increase in functional capacity, and a loss of 4.8 kg (p < 0.001) in body weight. No change took place during the control periods. Only those 7 patients who had had a five months long control period were included as controls in the present pooled analysis.

In the third study [1] 51 RA-patients concluded the trial. 26 tested a Cretan Mediterranean diet (MD) and 25 were controls. As a group the MD patients improved in 9 out of 14 disease outcome measures and lost 3 kg (p < 0.001) in body weight.

### The pooled patient material

Number of patients from the three studies is 95. Seven of these patients were studied in a cross-over design (2^nd ^study), and therefore, included both in the control and the intervention group giving a total number of 95+7. The patient demographics are shown in Table 1.

### Diets

The diets tested were lacto-vegetarian [3], strictly vegetarian, vegan [4] and a modified Cretan Mediterranean diet [1].

### Measurements

Five different measures of RA disease activity were identified, that had been assessed in all three studies. Two had been identically assessed in all three studies. These were the ESR (the Westergren erythrocyte sedimentation rate), and the Pain Score, which was the patient's self perceived pain severity as evaluated on a visual analogue scale (VAS, 0–100 mm).

The three measures, which were not similarly assessed and therefore not directly comparable, were firstly the blood plasma Acute-Phase Response [10], which in the 1^st ^study was measured with the reaction in plasma concentration of orosomucoid, and in the 2^nd ^and 3^rd ^with the corresponding reaction of C-reactive protein (CRP). The second was Physical Function as evaluated and recorded by the patient with a graded questionnaire of difficulties in performing specified tasks of daily living. A locally constructed questionnaire was used in the 1^st ^study, a not validated Swedish version of the Stanford-health assessment questionnaire (HAQ) in the 2^nd ^study, and the official Swedish version of HAQ [11] in the third study. The third dissimilarly assessed outcome measure was the Tender Joint Count [2], which in the 1^st ^study was the Ritchie joint index [12], and in the 2^nd ^and 3^rd ^study was the number of tender joints from palpation of 40, and 28 peripheral joints, respectively.

Due to different techniques of assessing these last three measures, change (Δ) obtained with these variables from baseline to the end of the experiments, could not be directly compared from the numerical results. These changes had to be converted into dichotomous values to enable a relevant statistical analysis. Change from baseline to the end of each study was thus characterised as improvement, or no improvement. These dichotomous variables are here called: dichoΔAcute-Phase Response, dichoΔPhysical Function and dichoΔTender Joint Count.

### Tested variables

#### Patient demographics

Gender, age and disease duration were used as independent variables. Age was characterised with respect to the median value, dichotomously, as <56 yrs or ≥ 56 yrs (dichoAge). Similarly, disease duration was characterised as <9.5 yrs or ≥ 9.5 yrs (dichoDisease Duration). Also the diet intervention was handled as an independent variable, and was characterised dichotomously, either as Case (lacto-vegetarian, vegan diet and Mediterranean) or as Control. The only independent outcome variable was the difference (Δ) from baseline to end of the study in body weight, and was characterised dichotomously as reduction or no reduction (dichoΔBody Weight).

#### Disease outcome variables

For evaluating effects that the dietary interventions might have had on RA disease activity, the difference (Δ) from baseline to end of study, in each of the five disease measures was calculated. For ESR and for Pain Score this was done numerically (numΔESR and numΔPain Score) but also, in the logistic regression, dichotomously characterised as improvement, or no improvement (dichoΔESR and dichoΔPain Score, respectively).

The other three RA disease measures were only handled dichotomously. They were: dichoΔAcute-Phase Response, dichoΔPhysical Function and dichoΔTender Joint Count, and were characterized as improvement /no improvement.

### Statistics

Baseline characteristics of the two diet groups were tested with non parametric tests (Mann-Whitney's U-test, and for gender Fischer's exact test; Table 1). Student's t-test and Mann-Whitney's nonparametric U-test were used to analyse group differences of change in numerical values of ΔESR and ΔPain Score, respectively.

The dichotomous variables were only analysed with logistic regression. All independent variables considered potentially significant were initially included in the model followed by a step-wise deletion of the least significant variable until a significant level of 0.05 or less. The statistical analyses were made using a commercially available computer programme (STATISTICA, StatSoft^®^, Tulsa, USA) and a p-value less than 0.05 was considered statistically significant.

## Results

### Baseline values before diet intervention

At baseline, no significant differences in Age, Gender, Body Weight or Disease Duration were found between the two groups (Table 1) or in the disease measures ESR and Pain Score.

### Change obtained from baseline to the end of the diet intervention period

#### Univariate analysis

Gender, Age, or Disease Duration were not associated with any of the outcome variables (p-values 0.2–1.0; univariate logistic regression; Table 2). The diet regimens rendered the diet group a weight fall of on average 3.5 kg, which is highly significant compared to the controls, who lost 0.1 kg (p < 0.001; Student's t-test). Body Weight reduction was univariately only correlated to improvement in dichoΔAcute-Phase Response (Table 2). Of the other outcome variables, ESR raised from 30.7 +/- 22 to 31.5 +/- 23 for the diet group and from 34.1 +/- 21 to 36.1 +/- 27 for the control group. These changes were not statistically significant (Student's t-test or Mann-Whitney's U-test) and did not correlate significantly with ΔBody Weight or dichoΔBody Weight. These findings were in accordance with the univariate logistic regression using the dichotomous variables (Table 2).

**Table 2:** Univariate logistic regression results (Odds Ratios (OR) and corresponding p-values), showing the associations between Gender, Age, Diet, Disease Duration and ΔBody Weight on the one hand, and the five different dichoΔ outcome variables on the other. Bolded values are statistically significant.

	Independent variable														
	
OR	Gender			Age (yrs)			Diet			Duration (yrs)			ΔBody Weight		
Effect variable	M	F	p	<56	≥56	p	Control	Case	p	<9.5	≥9.5	p	<0	≥0	p

dichoΔESR	1.00	1.02	0.98	1.00	1.22	0.62	1.00	1.29	0.55	1.00	1.00	1.00	1.00	1.64	0.29
dichoΔAcute-PR	1.00	0.60	0.33	1.00	0.74	0.45	1.00	**3.27**	**0.007**	1.00	1.17	0.69	1.00	**2.85**	**0.03**
dichoΔPain Score	1.00	0.47	0.18	1.00	0.65	0.29	1.00	**3.43**	**0.004**	1.00	1.27	0.55	1.00	2.10	0.10
dichoΔPhysic. F	1.00	0.86	0.78	1.00	0.74	0.45	1.00	**4.22**	**0.002**	1.00	1.38	0.42	1.00	2.16	0.10
dichoΔTender JC	1.00	0.93	0.90	1.00	1.54	0.28	1.00	1.54	0.29	1.00	1.22	0.62	1.00	1.77	0.20

Footnotes to table:

ESR = The Westergren erythrocyte sedimentation rate

Acute-PR = The Acute-Phase Response (Orosomucoid or C-reactive protein (CRP))

Pain Score = the patient's self perceived pain severity as evaluated on a visual analogue scale

Physic. F = Physical Function assessed by self completed questionnaires on the degree of difficulty in performing specified tasks of daily living

Tender JC = Tender Joint Count = number of painful joints at rest with pressure.

ΔPain Score showed a decrease of 10 units for the diet group and an increase of 2 units for the control group. This difference between groups was statistically significant (p = 0.011; Mann-Whitney's U-test) and was confirmed with the dichoΔPain Score difference (p = 0.007) (Table 2).

Using logistic regression, there were statistically significant correlations between diet and the results from three outcome variables. These were the dichoΔAcute-Phase Response, the dichoΔPain Score and the dichoΔPhysical Function, but not with the dichoΔESR or the dichoΔTender Joint Count (Table 2).

#### Multivariate analyses

The significant correlations using multiple logistic regression were limited to the association of dichoDiet to the three outcome variables dichoΔAcute-Phase Response, the dichoΔPain Score and the dichoΔPhysical Function, but not to the dichoΔESR or the dichoΔTender Joint Count (Table 3). Hence, body weight reduction (dichoΔBody Weight) was not statistically significantly coupled to any outcome variables when diet was taken into account.

**Table 3:** Multiple logistic regression results (Odds ratios (OR) and 95% confidence intervals(95% CI)), showing the association between the only statistically significant independentvariable, diet on the one hand, and the three disease outcome variables, which showedstatistically significant correlation to type of diet, on the other.

	Control diet			Intervention diet				
	
	Imp/no imp	Imp (%)	OR	Imp/no imp	Imp (%)	OR	95% CI	p
	
dichoΔAcute PR	12/30	29	1.00	34/26	57	3.27	1.39 – 7.67	0.007
dichoΔPain Score	17/25	40	1.00	42/18	70	3.43	1.49 – 7.93	0.005
dichoΔPhysic. F	10/31	24	1.00	34/25	58	4.22	1.73 – 10.3	0.002

Footnotes to table:

ESR = The Westergren erythrocyte sedimentation rate

Acute-PR = The Acute-Phase Response (orosomucoid or C-reactive protein (CRP))

Pain Score = the patient's self perceived pain severity as evaluated on a visual analogue scale

Physic. F = Physical Function assessed by self completed questionnaires on the degree of difficulty in performing specified tasks of daily living.

## Discussion

The time intervals between the 1^st ^and 2^nd^, and between the 2^nd ^and 3^rd ^studies were approximately ten years. One of the authors was local clinical research coordinator for all three projects. The studies were conducted in three different regions of Sweden. However, the patient populations were very much alike, as all patients were recruited from similar clinical settings at the central out patient rheumatology clinic of each region.

In all three studies the patients had been aggressively treated pharmacologically according to the internationally recommended guidelines that were prevailing at the time of each study. As a consequence of the more efficient disease modifying drugs of the 1990ths, the arthritis activity was better controlled in the 3^rd ^study than in the two earlier studies. With all three studies the participating patients had showed active interest for the kind of diet that was under investigation. At the time of the first two studies, vegetarian diets were advocated by laymen, while in the 1990ths much attention was focused on Mediterranean diets. Although different, these three diets shared some common characteristics which we believe were of importance with respect to control of inflammation. Compared to ordinary western diets they contained less of saturated fats from meat and dairy products. They had more of fresh fruits and of green vegetables. The MD was also rich in fats from sea foods, which was not a feature of the two vegetarian variants.

Another consequence of the time difference between the three studies was that the RA measures were not completely identical. They had successively been changed in line with the international recommendations of good scientific standards. Thus, comparable absolute baseline estimations of pain-score, acute phase response, physical function or tender joint count were not available due to their different definitions during the three intervention studies. Nevertheless, as explained in "methods", for the present analysis the outcome results of these variables were well defined, as they were dichotomously characterised as improvement, or no improvement.

The cross over design of the 2^nd ^study, with 7 patients assessed first as controls and later as diet patients, weakens the statistical power of the pooled statistical analysis only marginally.

In the three studies of ours each intervention i.e. with lacto-vegetarian, vegan or modified Cretan Mediterranean diet had rendered the patients a weight fall of on the average 2.4 kg over a trial length of 3–4 months. In the pooled analysis of change in body weight versus change in clinical outcome measurements the results were fairly clear. Apart from a univariate correlation between improvement in acute phase response and reduction in body weight, no statistically significant correlation was seen with the multiple logistic regression analysis between weight loss and the concomitantly obtained change in RA disease measurements. Thus the body weight loss seemed to have had no statistical significant anti inflammatory effect. However, although the number of patients was increased from pooling three different studies, the total amount of data was still relatively small. Therefore a possible anti inflammatory effect of weight reduction should not be completely discarded. Furthermore, the potential effect that weight reduction may have, should ideally be studied by itself, i.e. the test and control diets should differ only with respect to their contents of energy.

As for comparison of diet intervention versus control diet, the multiple logistic regression analysis, showed highly significant statistical correlations between diet intervention and improvement in RA outcome variables. These data indicate that dietary factors may have a potential role in treatment of RA. With regards to vegan diets, our observation is supported by the results of at least two independent, randomised and controlled studies [6,9].

Of course, the beneficial factors that the tested diets share need to be identified. A noteworthy candidate is their relatively low content of saturated fats [13]. Another is their high content of fresh fruits and vegetables.

Patients with active RA tend to have an abnormally increased peripheral insulin resistance [14]. Deliberate weight reduction in a group of patients with gout [15] was accompanied by reduction of insulin resistance and less numbers of inflammatory events. Whether this strategy for gout would work for patients with RA, and improve their arthritis, is an important clinical question. Some recent observational data indicate that overweight and obesity might be a risk factor for RA [16]. From experimental studies in mice [17] it is known that long-term pure energy under-nutrition has anti-inflammatory effects. In humans, deliberately undertaken short term fasting is well known to induce immune suppression and improvement in RA disease activity [18]. There are no controlled long-term studies of reduced energy intake without mal-nutrition on patients with RA. However, Iwasahige et al. [19] recently conducted a regiment for 54 days of caloric restriction combined with fasting in ten patients with RA. The patients lost in weight, and interestingly, the composite disease activity score of Lansbury was significantly reduced.

We know of two other small studies on deliberately undertaken weight reduction in patients with RA. In an uncontrolled pilot study [20] with 19 overweight patients with RA, Danish researchers had instructed the patients to lower their energy intake by 30% to achieve weight reduction. After a period of 12 weeks, the mean weight loss was 4.5 kg. No change was obtained in joint pain, morning stiffness, number of tender joints, or in sedimentation rate. Neither did Gordon et al. from their uncontrolled pilot study report any short-term favourable effects from assisting obese RA patients to reduce their body weight [21].

In conclusion, it seems as weight reduction strategies have little if any influence on RA inflammation. Perhaps this is not surprising with regards to what is already known from research on the metabolic syndrome and on atherosclerotic vascular disease, where isolated overweight is rank as a less important factor of risk [22].

Before conclusion of this discussion we need to remember that there is a controversy in letting patients with RA test an experimental regiment, which would involve prolonged reduction of their energy intake. During the course of RA most RA patients including those with overweight will develop a muscle wasting condition known as rheumatoid cachexia [22]. Although not directly fatal, this form of cachexia is believed to contribute to co-morbidity and reduced life expectancy. It is caused by the rheumatoid inflammation by itself and is refractory to nutritional therapy.
